# Observations on the Oriental Plague and on Quarantine, &c. Addressed to the British Association of Science

**Published:** 1839-07

**Authors:** 


### ART. XIII.

*Observations on the Oriental Plague and on Quarantines, &c. addressed to the British Association of Science.* By JOHN BOWRING. *Edinburgh,* 1838. 8vo, pp. 45.

IN noticing this pamphlet it is not requisite that we should enter into any discussion of the doctrines of contagion, or of the host of annoyances in the shape of quarantine regulations, lazarettos, inquisition of private documents, &c. to which merchants and travellers in the East are variously subjected in consequence of their reception. Dr. Bowring's statements, although worthy of every credence as far as his own personal observations are concerned, have yet too much the appearance of *ex parte* evidence, and his arguments too much of the character of special pleading, to justify the foundation of any very decided conclusions upon them adverse to existing laws and opinions. It cannot, however, be denied, that a strong case is made out for instituting a close and sifting enquiry : this, to be effective, must be carried on by candid and unbiassed observers in the centre of the plague districts; and it is but justice to the author to state, that to obtain an investigation of this description, is the professed object of this treatise.

Dr. Bowring's observations may be considered, first, in relation to the plague itself, and the quality of the evidence by which the doctrine of its contagious nature is supported; and, secondly, in relation to the efficiency of quarantine regulations in preventing or limiting the progress of the disease.

Considering the loose nature of the testimony upon which popular belief is often founded, it cannot be a matter of surprise that Dr. Bowring should find "much of the evidence floating about in the public mind as to the contagiousness of the plague to be of a very untrustworthy character," or that many "extraordinary absurdities," many "amusing inventions" have been resorted to, to account for the outbreak of the disease where no visible means of its introduction into a secluded locality present themselves. This is in strict accordance with what daily passes under our own inspection in many other diseases, and in some in which the specific contagion is of a far less questionable character than that of the plague. We believe that, were the medical records of small-pox to be investigated with a view to this point, some absurdities quite as extraordinary, some inventions quite as amusing, would be found brought forward to account for the outbreak of this latter disease, as the following instances adduced by the author with respect to the introduction of plague. "A very timid person," says Dr. Bowring, "an alarmed contagionist, who was attacked and died of the plague, had shut himself up in his chamber ; it was found that his son had, for his amusement, let up a kite from the roof of the house, and it was supposed that the kite-string had been touched by a bird, which bird was imagined to come from the infected quarter of the city (Alexandria or Cairo, it is not stated which); the plague entered the house down the string of the kite, and the son's father became the victim." "In another case, where the plague penetrated a house kept in the strictest quarantine, a cat had been seen to spring into a basket of clothes returning from the wash-house, and thence to leap into the window of the house in question. It was said the clothes belonged to some family which had probably had the plague; but, at all events, the cat was the only intruder who had violated the cordon, and was therefore the introducer of the disease. In a third instance," continues Dr. Bowring, "an Arab girl had hung a shirt out of a window to dry; the plague attacked the house, and I was told there could be no doubt that somebody in passing the street had touched the shirt, and was thus the cause of the introduction of the malady." But it is useless to multiply examples: it is perfectly obvious that the Arab girl had the means of communication with the street; and with the temptations to violate quarantine, and the natural heedlessness of individuals, especially of the ignorant, it is impossible to say what extent of communication might not have taken place. Instances of this description, as it appears to us, prove nothing, unless we are placed in possession of the fullest details of the precautions adopted, of the nature of the localities, and of the history of the persons inhabiting them.

A much stronger ground of objection to the received opinions of the contagious nature of the plague is afforded by the numerous instances in which, notwithstanding the abandonment of every precaution, both in respect of individuals and families, the disease has not been communicated from infected persons to those around. Several striking examples of this kind, occurring both during the general prevalence of the disorder and at times when only sporadic cases were observed, are related by Dr. Laidlaw of Alexandria, in a letter to Dr Bowring. It is not necessary to quote any of these in this place, but the following observation is too important to be passed over. "I have never seen," says Dr. Laidlaw, "a case of plague occurring sporadically where any person about the patient or in contact with him was attacked; and I cannot find any one that has *seen* one, although it is talked of among the Levantines as a common occurrence." The instances of inoculation with the blood or pus of plague patients, in which no effect followed, we are not disposed to place much reliance upon, as they are not only too few in number to warrant any conclusions being drawn from them, but the results obtained from this practice, upon the whole, were variable and uncertain.

We must refer to the pamphlet itself for the further elucidation of this part of the subject, and more especially for the arguments in favour of the endemic and epidemic character of the disease, derivable from its prevalence and fatality in low or close and confined situations, and from its entire cessation or altered character consequent upon the removal of the inhabitants into more healthy localities, or the scattering of the population over a greater extent of country. We proceed now to give the author's opinions as to the utter inefficiency of quarantine regulations for the attainment of the objects for which they are adopted.

Certainly, if we are to take up with the ultra views of many of the directors of quarantine establishments, the question as to the efficacy, not only of the existing laws in preventing the admission of plague, but also of any which may be hereafter devised, is reduced within very narrow limits. No quarantine, as Dr. Bowring observes, "can prevent the fish from passing up and down the Danube," or the bird from winging its flight through the air. It is not, however, by directing the shafts of ridicule against such absurdities as ignorant and interested individuals will always be found ready, from superstition and self-will or for special purposes, to advance and maintain, that the question can be set at rest. Rational evidence of the actual fact of quarantine inefficiency, as established by experience in places where every precaution has been used, must be brought forward, and the impossibility of providing against the existing defects shown, before the abstract principle of the inutility and consequent injustice of quarantine can be admitted.

One of the leading objections to the efficacy of quarantine regulations is, that wilful or interested persons will always find the means of evading them; and it must be acknowledged that in countries where there is a great extent of frontier to be protected, it seems scarcely possible to keep up a sanatory cordon in such a state of efficiency as to exclude all unauthorized communication. "Can the strictest quarantine," asks the author, "interdict the Arab of the desert from wandering where he will? Will the adventurous Khurd, the migratory Turkoman, the money-seeking Hebrew, the fanatical pilgrim, the potent sheik, be stopped in their peregrinations by the intervention of quarantine? In the universal system of corruption and bribery which exists in the East, can any functionary be depended on for imposing and enforcing a perfect obedience to sanatory regulations, and anything less than this is nugatory?" There is too much reason to fear that this is a just view of public or general quarantine. It is in accordance likewise with what has been observed elsewhere, and in the instance of diseases unquestionably of a highly contagious nature. Dr. Otto of Copenhagen, himself not opposed to quarantine regulations, has made the remark, that the abolition of the quarantine laws in Denmark in regard to smallpox has not caused any spreading of the disease; but, on the contrary, since the temptation to concealment has been thus removed, one cause of its more ready communication from individual to individual is done away with. But, according to Dr. Bowring, seclusion under private restrictive regulations is neither more easy of attainment nor more effectual than the general quarantine. "During the plague of 1835, the harem of the Pacha of Egypt consisted of about 300 persons; notwithstanding the severest cordon, the plague entered, and seven persons died within the cordon. The cordon itself was composed of 500 persons; these were in constant contact with the town where the plague was violently raging, and of these 500 only three died." This and similar instances, at the same time that they show the inefficiency of existing quarantine regulations entirely to exclude the pestilence, could it be shown that no infringement of the quarantine had taken place, would go far to prove that we must seek for other causes actively operating in the production of plague besides contagion.

This, however, by no means establishes the principle, that all sanatory measures should be thrown aside. A disease which originates from malaria, or other miasmata, may yet be capable of propagation by contact or otherwise from person to person, and it still remains a question of vital importance to determine the best mode of limiting the diffusion of such a disease. Whether the plague be considered as a purely contagious disorder, as endemic, and at the same time contagious, or as purely endemic, this question still remains; if the last of these views be correct, there can be no doubt that quarantine in every shape must be not only inefficacious, but fatally injurious in all its operations; if either of the former, in so far as the disease is contagious an effective quarantine must operate to the advantage of those who are secluded from infected persons. Whether a removal of the inhabitants from an infected district, and the scattering of them over a more extended surface in a healthy locality, might not in every case be more effective than any system of seclusion whatever, is a subject for future investigation. Some facts mentioned by Dr. Bowring, as well as others which have come under our own notice, in the malignant cholera and the continued fever of this country, would seem to countenance the affirmative of this question.

However this may be, it is quite clear, if we are to receive Dr. Bowring's statements, that in the article of fomites little or no precaution is taken,—bedding, blankets, clothing, cotton, and others of the most suspected articles, which are known to have been in contact with the persons of those infected with plague, being received on board of ships or not, according to the opinions, caprices, or interested views of those concerned; and, as it would seem, without injurious consequences.

We conclude these observations with quoting an instance of the inefficacy of the system of seclusion, which, but for the fatal termination, would be ludicrous in the extreme. "Among the medical men who were in Egypt, during the great plague of 1834-5, opinions were about equally divided as to the contagiousness or non-contagiousness of the disorder. But it is remarkable that, while only one of the non-contagionists died, several of the contagionists were victims. Those who took the greatest precautions were among the sufferers. M. Lardoni was a remarkable instance. He was the most timid of men; he never visited his patients but on horseback, and his appearance is thus described: "His harness was wholly of unsusceptible materials, his saddle closely covered with oilcloth, his stirrups were braided, and his reins made with filaments of the date tree; he had a huge oilskin cloak in the shape of a sack, which rose above his head and descended beneath his feet; he was always escorted by four servants, one before, one behind, and one at each side, so that no person could approach him. A thousand other ridiculous precautions were adopted by him; they were all in vain; he was attacked, though, for two days after the attack, he declared it was impossible it should be the plague; on the third he announced that it was really the dreaded calamity, and died soon after."

